# Integrating molecular classification and micrometastasis in sentinel lymph nodes in endometrial cancer management

**DOI:** 10.1590/1806-9282.20260304

**Published:** 2026-08-10

**Authors:** Antonio Cassio Assis Pellizzon

**Affiliations:** 1 AC Camargo Cancer Center – São Paulo (SP), Brazil.

## INTRODUCTION

Endometrial cancer (EC) is the most common gynecologic malignancy in developed countries. The American Cancer Society estimates for 2026 about 68,270 and 14,450 new cases and deaths, respectively, related to endometrial cancer^1^. In Brazil, there are a projected 9,500 new diagnoses in the same year^2^.

EC arises from the glandular epithelium of the endometrium and is therefore histologically classified in most cases as an adenocarcinoma. Tumor dissemination commonly follows the uterine lymphatic drainage pathways, including pelvic lymph nodes—particularly obturator, internal iliac, external iliac, and common iliac stations—and, in higher-risk disease, to para-aortic chains^3^. This close relationship between the adenocarcinoma origin of endometrial tumors and their characteristic lymphatic dissemination pattern underpins the rationale for nodal evaluation through imaging, sentinel lymph node mapping, and tailored radiotherapeutic or surgical approaches.

Lymph node dissemination is a crucial factor for the patient's prognosis. The transition from systematic lymphadenectomy to sentinel node mapping (SLN) has led to more sensitive detection of low-volume nodal disease. Ultrastaging techniques now identify micrometastases and isolated tumor cells (ITCs) that were previously undetected^4^.

According to the American Joint Committee on Cancer and following the Philadelphia Consensus Conference on sentinel nodes in breast cancer, definitions have been proposed: macrometastases as a single focus of metastatic disease per node measuring more than 2 mm; micrometastases as a focus of metastatic disease ranging from 0.2 mm to no more than 2 mm^5^ and; in accordance with Marchiolé et al., submicrometastases as metastases measuring no more than 0.2 mm, including the presence of a single non-cohesive tumor cell^6^.

Micrometastases are considered true nodal metastases and upstage patients to stage IIIC disease. However, their prognostic weight appears intermediate between node-negative and macrometastatic disease^5^.

The FIRES (Fluorescence Imaging for Robotic Endometrial Sentinel lymph node biopsy) trial and subsequent validation studies established SLN mapping as an oncologically safe alternative to full lymphadenectomy. Micrometastases can be detected in approximately 5–15% of patients, and many of these cases would have been classified as node-negative with conventional histopathology^7^. This stage migration has important therapeutic consequences, particularly for adjuvant therapy decisions.

Multiple retrospective analyses suggest that micrometastasis is associated with increased recurrence risk compared to node-negative disease. However, disease-free survival (DFS) is generally superior to that of patients with macrometastases, but the absolute increase in pelvic recurrence is modest when adjuvant therapy is administered^8^.

Despite decreased DFS, the impact on overall survival remains inconsistent across studies^9,10^.

Recently, the integration of molecular subgroups has transformed risk stratification. While DNA Polymerase Epsilon, Catalytic Subunit (POLE)-mutated tumors demonstrate an excellent prognosis, potentially mitigating the adverse significance of micrometastasis, the p53-abnormal tumors exhibit high systemic risk, where even low-volume nodal disease may indicate aggressive biology, and mismatch-repair-deficient and no specific molecular profile tumors show intermediate behavior^11,12^. Thus, micrometastasis must be interpreted within the molecular context rather than as an isolated pathologic finding.

Micrometastasis generally supports escalation beyond vaginal brachytherapy alone. Most contemporary guidelines recommend systemic therapy consideration and regional nodal irradiation, particularly when additional uterine risk factors are present^13^. The key clinical question is not whether micrometastasis is relevant, but rather how aggressively regional nodal basins should be treated.

For radiation oncologists, micrometastasis is treated as node-positive disease. However, field design must balance the regional control, distant relapse risk, and acute and late treatment morbidity. For most patients with pelvic SLN micrometastasis, the whole pelvic radiotherapy is recommended, encompassing common, external, and internal iliac nodes, obturator nodes, and the presacral region. The standard dose ranges from 45 to 50.4 Gy, and, importantly, micrometastatic nodes rarely require a radiation boost unless gross residual disease is suspected. Modern intensity modulated radiation therapy/volumetric modulated arc therapy techniques are recommended to minimize toxicity.

The vaginal cuff brachytherapy may be added depending on uterine risk factors. Limiting treatment to vaginal brachytherapy alone is generally discouraged unless all of the following are present: low-grade endometrioid histology, no lympho-vascular invasion present, a favorable molecular subtype, and /or systemic therapy administered^14^.

Differently from micrometastasis, ITCs (≤0.2 mm) are classified as pN0(i+) and may not carry the same prognostic impacts. Current evidence suggests ITCs may represent an early form of nodal involvement, biologically distinct from micro- and macrometastases. ITCs alone may not mandate pelvic nodal irradiation, particularly in low-risk patients^15^.

While macrometastatic nodal involvement is clearly associated with worse oncologic outcomes and guides adjuvant treatment recommendations, the prognostic and therapeutic implications of micrometastases and ITCs are less well defined. Some studies suggest that low-volume nodal disease may represent an early stage of lymphatic dissemination associated with increased recurrence risk, whereas others report outcomes comparable to node-negative patients, particularly in the absence of additional uterine risk factors^16^. Consequently, uncertainty persists regarding whether these findings should systematically prompt treatment escalation with chemotherapy and/or radiotherapy. The increasing incorporation of molecular classification further complicates this issue, as the biological behavior of the primary tumor may outweigh the prognostic impact of minimal nodal involvement. Therefore, the management of micrometastases and ITCs continues to be an area of active investigation, with ongoing efforts aimed at integrating pathologic, molecular, and clinical features to better define their true prognostic relevance and optimize individualized treatment strategies.

The future urges prospective data to clarify the independent prognostic value of micrometastasis, define the role of de-escalation in POLE-mutated tumors, integrate molecular classification into RT volume design algorithms, and determine the optimal balance between systemic therapy and regional irradiation. Ongoing trials incorporating molecular risk stratification will likely refine indications for extended-field irradiation.

## CONCLUSION

Micrometastasis in sentinel lymph nodes represents biologically meaningful nodal spread in endometrial cancer. It upstages patients to stage IIIC and generally warrants pelvic nodal irradiation in combination with systemic therapy when additional risk factors are present, providing the indication for further adjuvant treatment.

Incorporating the biological behavior of the tumor in addition to histologic subtype and stage has become increasingly important in guiding individualized treatment decisions, including the extent of nodal assessment, indications for adjuvant radiotherapy or chemotherapy, and the identification of patients who may benefit from treatment de-escalation or intensified multimodality therapy.

In the modern era, radiotherapy planning for micrometastatic disease should shift from a purely anatomic paradigm to a biology-informed strategy, optimizing oncologic control while minimizing treatment-related morbidity.

## Data Availability

The datasets generated and/or analyzed during the current study are available from the corresponding author upon reasonable request.
